# The Effect of Altitude on Ocular Functions

**Published:** 1919-01

**Authors:** 


**The Effect of Altitude on Ocular Functions.** By W. H. WILMER, Colonel M. C., N. A., and C. BERENS, Captain M. R. C., U. S. Army. *The Journal of the American Medical Association.* October 26, 1918.

The authors speak of the importance of good vision in airmen and record the studies made on visual acuity under oxygen depletion, as compared with this function under normal oxygen supply. The results of visual tests carried out by means of the Yves visual acuity test object, Snellen's type and Johnson's apparatus, on men classified as normal and subnormal (i. e. those who would pass the examination for the F. C. and those who would be ocularly disqualified) are as follows:

| | Normal | | Subnormal. | |
|---|---|---|---|---|
| | No. | per cent. | No. | per cent. |
| Vision improved . . . . . | 3 | 12 | | |
| Vision deteriorated.. . . . | 7 | 28 | 3 | 37.5 |
| No change. . . . . . . . | 15 | 60 | 5 | 62.5 |

*Judgment of Distance and Stereoscopic Vision.* The stereoscopic vision was tested on the Henderson rebreathing apparatus and in the low-pressure chamber by the use of the ordinary stereoscope, and the ability to maintain perfect stereoscopic vision at different altitudes noted. In some cases, showing confusion at high altitudes, normal stereoscopic vision was restored by the administration of oxygen.

*Reaction Time.* The simple reaction time has been disqualified as too apt to become a reflex, and the Reeves visual discrimination reaction time experiment, with 4 possible reactions and 5 possible stimuli, chosen. The average discrimination reaction time for normal subjects has been found to be about half a second.

*Color Vision.* The effect of low-oxygen tension on color vision has been determined by means of Stilling's plates, Series of subjects were examined in the rebreathing apparatus and in the low-pressure chamber at an altitude of 20,000 feet and above. No changes were noted.

*Field of Binocular Fixation.* The field of binocular fixation has been taken by means of the Schweiger perimeter modified for binocular use or by means of a large tangent screen, the Wilmer wand and Wilmer glasses being used. Of 122 normal and 16 subnormal men examined, 7.37 o/o of the normal and 50 o/o of the subnormal showed contraction of the field of binocular fixation.

*Muscle Balance.* Normal muscle balance should be insisted upon, for even a small defect may, owing to the strain in flying and lack of oxygen, result in diplopia or at least in a marked contraction of the field of binocular fixation at low altitudes. Exophoria and hyperphoria are more serious than esophoria. 25 normal men were examined in the low-pressure tank and in the rebreathing apparatus; abduction decreased 1.31 and 1.75 degrees at 15,000 feet and 1.55 and 1.90 at 20,000 feet. Sursumvergence decreased 1.15 degrees at 15.000 and 1.25 degrees at 20,000 feet.

*Field of Vision.* The fields for form and color have been taken in the low-pressure tank at 5,000, 10,000, 15,000 and 20,000 feet; and when contraction was noted, at 20.000 feet, oxygen was administered. To make sure that the changes were not due to fatigue, controls were taken at sea level corresponding in time of day and time interval to those taken in the low-pressure chamber, At 5,000 and 10,000 feet there is usually slight enlargment of the fields for form and color, at 15,000 a slight contraction, and at 20,000 a marked contraction,

*Perception of Motion and its Direction by the Retina.* The test is performed with the subject seated 15 inches from a Bjerrum screen, fixing with both eyes a 3 mm. white pin placed in the screen on a level with the eye. A small light, which is visible through the cloth, is used as the test object. The point at which the test object is first noted, and where the direction of motion of 3 planes is first accurately described, is recorded on a chart for use with the tangent screen. In the men examined there was an average difference of 10 degrees between the time when the test object was first noted and the direction of motion accurately described.

*Intra-ocular Tension.* No correlation has been found between the intra-ocular tension and the blood pressure, lowered oxygen tension and various cardiovascular changes.

*Accommodation in Flying.* The near point of accommodation has been taken every minute on the rebreathing apparatus, a Prince rule with Jeager test type or the Duane disk being used as a test object. Of 148 normal men, 44.6 o/o showed a receding of the near point and 18 o/o showed improvement; fluctuating changes were noted in 14.4 o/o and no change in 23 o/o. The low-pressure chamber findings were practically the same. The inhalation of oxygen invariably causes a return to normal. Subjects with a marked amount of hyperopic astigmatism show the most marked changes in accommodation.

*Convergence.* A U-shaped piece was out cut of the Prince rule to fit over the nose, and a 2mm. black dot on a white ground was used as a test object for making the determination. Readings were taken without and with low oxygen tension effect, and the effect of administration of oxygen noted.

Readings were taken every 2 minutes and charted. The results obtained were identical both with the low-pressure tank and the rebreathing apparatus, and in each case the determining factor seemed to be the lowering of oxygen tension.

*Retinal Sensitivity.* The Reeves wedge calibrated in mms. has been used for the determination of the threshold sensitivity; the test of contrast sensitivity is made with a Reeves wedge and Reeves contrast square.

The conclusions drawn by the authors may be thus summarized: the changes in the eye are due mainly to the want of oxygen, not to the nervous strain, vibration of the motor, etc. The administration of oxygen prevents the occurrence of the untoward symptoms; but when these symptoms have occurred, oxygen quickly restores the functions to normal.

